# Sleep Characteristics, Sleep Problems, and Associations to Quality of Life among Psychotherapists

**DOI:** 10.1155/2012/806913

**Published:** 2012-06-11

**Authors:** Angelika A. Schlarb, Dorota Reis, Annette Schröder

**Affiliations:** ^1^Faculty of Psychology, University of Koblenz-Landau, 76829 Landau, Germany; ^2^Department of Psychology, Faculty of Science, University of Tübingen, Schleichstrasse 4, 72076 Tuebingen, Germany

## Abstract

Sleep problems, especially insomnia, are a common complaint among adults. International studies have shown prevalence rates between 4.7 and 36.2% for sleep difficulties in general, whereas 13.1–28.1% report insomnia symptoms. Sleep problems are associated with lower social and academic performance and can have a severe impact on psychological and physical health. Psychotherapists are suppliers within the public health system. The goal of this study was to outline sleep characteristics, prevalence of sleep problems, insomnia, and associations of quality of life among psychotherapists. A total of 774 psychotherapists (74.7% women; mean age 46 years) participated in the study. Sleep characteristics, sleep problems, well-being, life satisfaction and workload, as well as specific job demands, were assessed via a questionnaire. Analyses revealed that more than 4.2% of the surveyed psychotherapists have difficulties falling asleep, 12.7% often wake up in the night, and 26.6% feel tired, and 3.4% think that their interrupted sleep affects work performance. About 44.1% of them suffer from symptoms of insomnia. Path models showed that insomnia is significantly related to well-being and life satisfaction.

## 1. Introduction

Sleep is fundamental for physical and emotional recovery. Therefore, sleeping well is essential to overcome successfully daily hassles and to prevent long-term negative effects of stress on health. In general, health or good sleep seems to be very important for both physiological and psychological well-being.

Stress leads to psychological and physiological arousal, and, therefore, leads to impaired sleep [1]. Work overload is connected with several sleep difficulties, such as difficulty falling asleep, difficulty maintaining sleep, and nonrestorative, sleep [2]. Furthermore, people who perceive their work as hectic or exhausting suffer from disturbed sleep and fatigue significantly more often [3]. Stress due to an imbalance between effort and reward seems to be connected with sleep disturbances [4], whereas this association seems to be more common amongst men [5]. Linton showed that employees without sleeping problems at baseline had a twofold risk of sleep difficulties one year later, after having experienced stress, such as a poor psychosocial work environment [6]. Ota and colleagues showed that low social support and an effort-reward imbalance among insomniacs were significantly associated with insomnia at followup. On the other hand, overcommitment to work and high job strain among health persons at baseline were associated with insomnia at followup [7]. Hence, there is evidence for a relationship between stress at work and impaired sleep.

It has been taken into account that, to some degree, everybody is predisposed to develop insomnia. Often a precipitating event is connected with the onset of insomnia symptoms. Several influencing factors are associated with the onset and chronicity of insomnia symptoms, such as stressful life events (e.g., divorce) [8, 9], psychological and health-related factors [10], demographic factors (e.g., female gender, aging, living alone; [11]), psychological diseases such as depression and anxiety [12–14], as well as a personal or family history of insomnia [15, 16].

Prevalence rates of disturbed sleep overall range from 35% to 41% among adults [17]. Insomnia symptoms have prevalence rates ranging from 23% to 34% [18]. As Krishnan showed in her review, healthy women normally report better sleep quality compared with men. Furthermore, they report longer sleep times, shorter sleep onset latency, and higher sleep efficiency. However, it also has to be mentioned that women suffer from more sleep-related complaints than men [19]. Several studies have recorded a female predominance with regard to insomnia [20]. Also, Healey and colleagues reported that a new onset of insomnia is generally more frequent among women and individuals with a perceived stressful [8].

If a person suffers from poor sleep, his or her well-being is affected. Chronic sleep problems are associated with numerous complaints concerning social [21] and academic performance, such as difficulties with concentration [22], as well as concerning psychological and physical health [10], social dysfunction, and poorer self-rated health [23]. Suffering from insomnia corresponds with increased fatigue and decreased attentiveness and job satisfaction [24]. Coping strategies and problem-solving competence are factors that may partly account for the differences in health and sleep quality [10, 25–27]. Some research has been conducted to focus on positive emotions, such as experience of happiness or subjective well-being [28]. High satisfaction with life or, in more detail, with specific life domains such as job satisfaction and satisfaction with housing, and so forth, might be a resilience factor concerning sleep problems [29].

Only few studies exist concerning work stressors, personal health, and sleep in health care professionals. Studies of nurses and midwives show that their work is associated with higher rates of error at work [30, 31]. Regarding the profession of psychotherapists, the sample of this study, it can be stated as a crucial point that they have a fundamental responsibility for their patients. This includes—among other competences—to empathize with the human person. Therapeutic techniques or methods such as stimulus control or cognitive strategies have to be applied. In the process of therapy, he or she needs self-awareness and mindfulness, not only concerning the therapeutic alliance [32]. Finally, he or she needs to recognize the hazards of psychological practice [33] and be aware of the suicide potential of the patient. Hence, it is of essential interest to identify variables that have an effect on sleep quality.

To our knowledge, an investigation of sleep problems and quality of life among psychotherapists has not been conducted to date. The goal of this study is to close this gap by outlining sleep problems and prevalence of insomnia symptoms among psychotherapists. A further objective is to evaluate which factors predict insomnia in psychotherapists and answer the question as to whether insomnia mediates the impact of job strains on aspects of the quality of life.

## 2. Participants and Procedure

The data presented in this paper is part of an extensive two-wave survey, which mainly focuses on gaining knowledge of different aspects of occupational health such as well-being, burn-out, job-associated and personal resources, as well as demands among psychotherapists in Germany [34].

A total of 774 practicing therapists (mean age  46 ± 10.8  years) participated in an online questionnaire study. 74.7% were female, as this occupation is strongly preferred by women in Germany. The majority were registered psychological psychotherapists (69.0%), psychological psychotherapists in education (18.5%), and medical psychotherapists (3.9%), all of which had either a cognitive-behavioral or an analytic focus. The rest was split up into psychotherapists with other professions (alternative practitioners, social workers) and practitioners with qualifications such as systemic or hypnotherapy working in the field of mental health. Participants' mean work experience was 17.5 ± 10.8 years, while the average weekly working time was reported as 39.7 ± 11.4 hours.

## 3. Measures

### 3.1. Insomnia

Insomnia was measured with the German version of the Arabic Scale of Insomnia [35]. The ASI is a short questionnaire of 12 items complying with the diagnostic criteria of both the Diagnostic and Statistical Manual of Mental Disorders, Fourth Edition (DSM-IV), and the revised International Classification of Sleep Disorders (ICSD). Participants were requested to respond to each item on a 5-point scale labeled from 0 (= hardly ever) to 4 (= very often). Following Abdel-Khalek's [34] suggestion, the percentage of “3” (= often) and “4” (= very often) ratings was considered to be noticeable. The ASI has demonstrated good internal consistency with Cronbach *α* ranging between  .86 and  .87 and test-retest reliabilities ranging from  .70 to  .76 [35]. In the present study, the internal consistency amounted to  .84. The questionnaire has been applied due to affecting work performance.

### 3.2. Subjective Well-Being

Well-being was assessed with the German version of the WHO (Five) Well-being Index (WHO-5, WBI). The instrument contains five items which cover positive mood, vitality, and general interests. Each of the items is rated on a 6-point Likert scale from 0 (= not present) to 5 (= constantly present). WBI has well-established psychometric properties and seems to be a very adequate screening instrument for depression [36]. The coefficient *α* for the scale was found to be  .84 in this sample, confirming high internal consistency.

### 3.3. Satisfaction with Life

The Satisfaction With Life Scale SWLS [37] was used to assess the life satisfaction component of subjective well-being. Including only five items, the instrument has demonstrated good psychometric characteristics with the coefficient alpha ranging from  .79 to  .89, indicating high internal consistency [28]. The items can be answered using a 7-point Likert style response scale (ranging from 0 = strongly disagree to 6 = strongly agree). Scores ranging between 26 and 30 indicate extreme satisfaction with life, whereas scores between 0 and 4 demonstrate respondent's extreme dissatisfaction with life. A score of 15 represents the neutral point of the scale.

The SWLS has been utilized in numerous studies; the scores correlate with different measures of mental health (BDI, e.g., for an overview see [28]) and predict future behaviors (adjustment after medical procedures, e.g., [38]). In the present study, Cronbach *α* was found to be  .86.

### 3.4. Work Load

Work load was measured with three items retrieved from the German version of De Vragenlijst Beleving en Beoordeling van de Arbeid (VBBA, [39]). All items were scored on a 4-point scale ranging from 1 (= never/not at all) to 4 (always). A sample item of this scale is: “Do you have to work very fast?” Good validity and reliability of the VBBA have been demonstrated [40]. The Cronbach *α* coefficient for the three items used in the present study was  .68.

### 3.5. Specific Job Demands

Job demands specific for the occupation of psychotherapists were assessed with a specifically constructed scale. Twelve items were retrieved from empirical outcomes concerning strains in mental health and from qualitative interviews. Participants were asked “What are the most important demands of psychotherapeutic work for you?” Sample statements are “Feelings of threat by patients”, “Doubts about own therapeutic skills” or “Responsibility for endangerment to others or self-endangerment”. All items could be rated on a 6-point scale from 1 (= not at all) to 6 (= very much). The Cronbach coefficient *α* was found to be  .84, indicating good internal consistency.

## 4. Data Analysis

After determining the descriptive statistics for the scales, the first research question regarding the different personal and job-related attributes potentially influencing insomnia was addressed, applying univariate analyses.

In order to address the impact of insomnia on subjective well-being and life satisfaction, four different mediation models were examined. Baron and Kenny's approach [41] was applied to test the postulated mediation effects. According to this approach, a variable is considered a mediator if (1) the predictor is significantly associated to the outcome, (2) the predictor is significantly associated with the mediator, and (3) the mediator significantly predicts the outcome after controlling for the predictor [42]. Finally, a mediation effect can be determined when the relationship between the predictor and the outcome becomes significantly weaker (partial mediation) or non-significant (full mediation) after including the mediator. The Sobel test was conducted to examine the significance of the indirect effect. To avoid difficulties with the assumption of normal distribution under the null hypothesis, which is normally not given for the distribution of products, the sampling distributions of the indirect effects were bootstrapped to derive a reliable confidence interval.

SPSS 19.0 was used for these computations. Significance testing with a Sobel test as well as bootstrapping (5000 bootstrap resamples) was processed using the Sobel macro by Preacher and Hayes [42].

## 5. Results

Descriptive statistics for all 5 scales are shown in Table 1. 36.8% of the participants report poor subjective well-being (indicated if sum score 13 or below). On the other hand, they report typical life satisfaction scores above the neutral point [28] revealing a distinction between the two concepts. The insomnia scores turn out to be rather low. However, job demands, namely, workload and specific job strains, occur with higher frequency.

The occurrence of insomnia symptoms among psychotherapists is heterogeneous. First of all, they often report tiredness (26.6% answering “often” or “very often”), interrupted and disturbed sleep (14.4%), and waking up many times (12.7%). However, psychotherapists do not assume that their relationships or work performance are affected by sleep interruptions.  Table 2 shows the item characteristics and the prevalence of highly rated insomnia symptoms. 

Further results show that 44.1% of psychotherapists report at least one or more insomnia symptoms. More than 7% report four or more symptoms, whereas 55.9% do not suffer from any sleep problems (all data in Table 3). 

### 5.1. Personal and Job-Related Attributes Influencing Insomnia

In order to determine which personal and job-associated variables may influence insomnia, various demographic variables and job characteristics were related to the insomnia score. These univariate analyses indicated no significant associations between sociodemographic variables such as gender, age or family status, and insomnia. Furthermore, job characteristics such as job experience, working hours per week, and being in training versus being licensed also revealed no significant relationships with insomnia. Only higher incomes provided a low inverse correlation with insomnia symptoms (*r*(757) = −.08, *P* = .03).

### 5.2. Predictive Power of Insomnia on Subjective Well-Being

**Figure 1:**
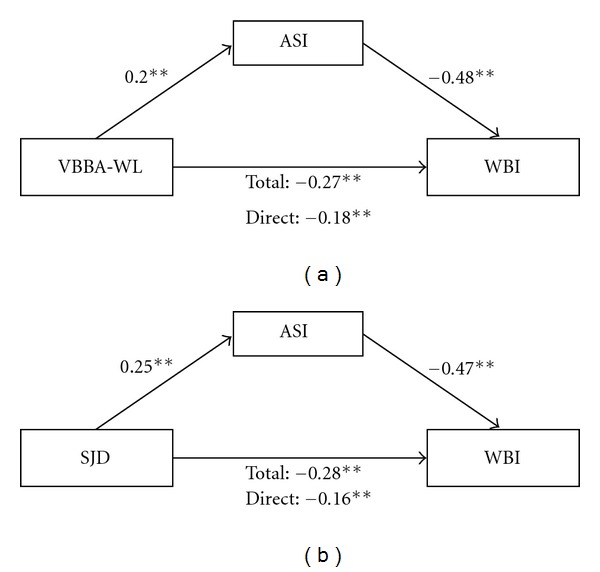
Path models for subjective well-being. Note. VBBA-WL: workload, SJD: specific job demands, ASI: Insomnia, WBI: well-being Index.

Before testing insomnia as a mediator of the relationship between job demands and aspects of quality of life, the three prerequisite conditions had to be verified. Results of a linear regression analysis showed that the workload as well as specific job demands were related to both general well-being (*β*_WL→WBI_ = −.27, *P* < .001; *β*_SJD→WBI_ = −.28, *P* < .001) and insomnia (*β*_WL→ASI_ = .20, *P* < .001; *β*_SJD→ASI_ = .25, *P* < .001). Furthermore, insomnia was related to well-being (*β*_ASI→WBI(WL)_ = −.48, *P* < .001; *β*_ASI→WBI(SJD)_ = −.47, *P* < .001) after controlling for job demands, making it possible to proceed with the mediation test. Therefore, all paths were statistically significant and in the expected direction. Both of the path models as well as the standardized path coefficients are shown in Figure 1. The addition of insomnia to the model reduced the magnitude of the total association between quantitative and qualitative job demands and subjective well-being significantly: for workload from *β* = −.27 to *β* = −.17, Sobel *z* = −5.39; *P* < .001; for specific job demands from *β* = −.28 to *β* = −.16, Sobel *z* = −6.29; *P* < .001. The effect sizes (completely standardized indirect effects) [43] for workload and specific job demands amount to −.16 (LL95CI: −.22, UL95CI: −.10) and −.094 (LL95CI: −.13, UL95CI: −.06), respectively, indicating medium effects. 

### 5.3. Predictive Power of Insomnia on Life Satisfaction

**Figure 2:**
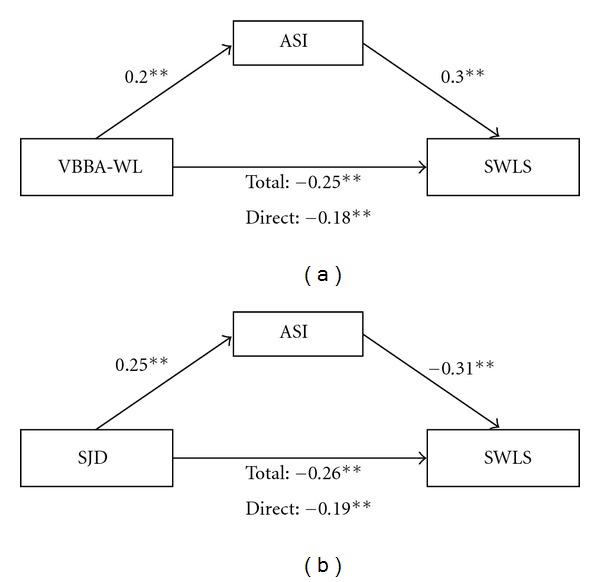
Path models for life satisfaction. Note. VBBA-WL: workload, SJD: specific job demands, ASI: insomnia, SWLS: life satisfaction.

In order to test insomnia as a mediator of the association between job demands and life satisfaction, the three prerequisite conditions had to be verified again. The outcomes of a linear regression analysis indicated that workload as well as specific job demands were again related to both life satisfaction (*β*_WL→SWLS_ = −.25, *P* < .001; *β*_SJD→SWLS_ = −.26, *P* < .001) and insomnia (*β*_WL→ASI_ = .20, *P* < .001; *β*_SJD→ASI_ = .25, *P* < .001). Furthermore, insomnia was related to life satisfaction (*β*_ASI→SWLS(WL)_ = −.30, *P* < .001; *β*_ASI→SWLS(SJD)_ = −.31, *P* < .001) after controlling for job demands, so that the assumptions of the Baron and Kenny approach were fulfilled. Therefore, all paths were statistically significant again as well as in the expected direction. The addition of insomnia to the model again reduced the magnitude of the total association between quantitative and qualitative job demands and life satisfaction significantly: for workload from *β* = −.25 to *β* = −.18, Sobel *z* = −4.83; *P* < .001; for specific job demands from *β* = −.26 to *β* = −.19, Sobel *z* = −5.44; *P* < .001. The effect sizes (completely standardized indirect effects) for workload and specific job demands amount to −.12 (LL95CI: −.17, UL95CI: −.07) and −.07 (LL95CI: −.11, UL95CI: −.04) respectively, indicating small and medium effects on life satisfaction.  Figure 2 shows both path models together with standardized path coefficients. 

## 6. Discussion 

The results of this study, based on data from 774 psychotherapists—reveal that sleep problems are quite common among these health care professionals: more than 44% of the therapists reported sleep difficulties. These rates are higher than those recorded by Ancoli-Israel and Roth (up to 34%; [18]). Moreover, according to our findings, insomnia (diagnosed via ASI) is associated with lower life satisfaction and well-being. This corresponds with other results that have shown that insomnia is associated with lower quality of life [44, 45]. Besides this result, one has to keep in mind that several insomnia instruments are available for adults and, therefore, the results might vary due to different instruments. Furthermore, Morin and colleagues also reported psychological and physical impairments resulting from insomnia [10]. In addition, the results of Elovainio et al. show poorer self-rated health as well as social dysfunction among insomniacs [23]. 

In our study, no gender effect was observed; this contrasts with other studies, which have shown that sleep difficulties are more common in females than in males [46, 47]. Urponen and colleagues also found that disturbed sleep was more frequent in women [48]. In general, women report more sleep problems, for example, inadequate sleep time or insomnia [49]. 

Normally, age is a widely discussed predictor of disturbed sleep [50]. Indeed, higher age was shown to be a clear risk indicator for disturbed sleep in Åkerstedt's study [3]. In addition, Zhang and Wing showed in their study that the risk of insomnia in women compared with men increases with age [49]. In contrast with those results, our study showed that sleep problems in psychotherapists do not differ with regard to age. 

Ancoli-Israel and Roth [18] as well as Urponen and colleagues [48] have demonstrated that occupational stress is correlated with sleep disturbances. Moreover, people with sleep disturbances attribute their sleep problems to occupational stress. Our results have shown that a high workload is associated with insomnia. This is in line with the findings of Ribet and Derriennic, who found in a large cohort study of 21,000 subjects that shift work, a long working week, exposure to vibrations, and hurrying at work appeared to be the main risk factors [51]. However, Åkerstedt suggested that work demands are not only the main predictor, but rather their effect on postworking time [3]. Results have also shown that workload was related to insomnia, which is in line with the results of other researchers. Indeed, workload ratings were a significant predictor of extreme tiredness/exhaustion in a study among rail industry workers [51]. Moreover, a recent study by Lallukka and colleagues showed that both physical and psychosocial working conditions were strongly related to sleep complaints [52]. 

Resilience factors for insomnia also have to be considered. Previous results of our research have shown that personal resources, autonomy, and learning opportunities were named by the therapists as factors influencing job efforts [34]. These factors could be discussed as resilience factors concerning sleep difficulties; this is in line with Kuppermann and colleagues who have shown that subjects who are satisfied with work report less sleep problems than persons struggling with their jobs [53]. Furthermore, mental effort at work seems to predict disturbed sleep better than any objective indicators [54]. 

Several limitations have to be named. First, other factors (e.g., arousal, anxiety, or medical illnesses) could also be responsible for the perception of work stressors and for insomnia. Second, additional information as smoking, physical activities, as well as caffeine use or medications were not collected due to other aims of the survey. 

Third, long-term outcomes are not available; therefore, future research should investigate the role of work stressors on the development and maintenance of insomnia. Fourth, all data were assessed in a cross-sectional way and without objective measurements. Thus, it is not possible to answer the question as to whether sleep symptoms, especially insomnia, are a precursor to or a consequence of workload or mental health problems. In the future, objective data are needed. 

All in all, the results of this study demonstrate that stressors are also related to insomnia in psychotherapists' psychosocial work. Furthermore, the findings of this study indicate that some workload and specific job demands are associated with insomnia. Moreover, insomnia is negatively related to psychosocial well-being and life satisfaction. On the other hand, factors such as age or gender seem to be less substantial. In conclusion, insomnia has to be taken into account when discussing stress at work, workload, and job demands. As the results suggest psychotherapists face similar problems as other professions and, therefore, should not be seen as resistant to sleep difficulties.

## Figures and Tables

**Table 1:** Descriptive statistics for scales.

Scale	Theoretical range	*M*	SD	skewness
ASI	0–4	.70	.57	1.12
WBI-5	0–5	2.94	.90	−.50
SWLS	0–6	4.17	1.04	−.84
VBBA-WL	0–3	1.32	0.55	.54
SJD	1–6	2.79	1.08	.23

Note. ASI: Arabic Insomnia Scale, WBI-5: Well-being Index, SWLS: life satisfaction, VBBA-WL: three workload items derived from VBBA, SJD: Specific Job Demands Scale.

**Table 2:** Prevalence of insomnia symptoms among psychotherapists.

Item	*M*	SD	Percentage highly rated symptoms
(1) Difficulties falling asleep	.51	.87	4.2
(2) Interruption and disturbance	1.01	1.17	14.4
(3) Waking up many times	.92	1.10	12.7
(4) Waking up early	.75	1.05	9.4
(5) Feelings of depression at bedtime	.41	.79	3.4
(6) Bad thoughts before falling asleep	.37	.65	6.2
(7) Tired when waking up	1.66	1.19	26.6
(8) Waking up in a bad mood	.52	.87	4.5
(9) Becoming tense when waking up	.65	.89	5.2
(10) Interrupted sleep is annoying	.63	.98	7.8
(11) Interrupted sleep affects relationships	.33	.73	2.9
(12) Interrupted sleep affects work performance	.62	.84	3.4

Note. All items from ASI. Percentage of response options 3 and 4 (i.e., “often” and “very often”).

**Table 3:** Percentage of highly rated insomnia symptoms among psychotherapists.

Number of symptoms	*N* (%)
0	433 (55.9)
1	168 (21.7)
2	72 (9.3)
3	42 (5.4)
4 and more	59 (7.7)
